# A modified HIV model with Beddington–DeAngelis incidence and cure rate

**DOI:** 10.1038/s41598-026-47946-0

**Published:** 2026-05-26

**Authors:** Sarah Ramadan, Sanaa Salman, Ahmed EL-Sayed

**Affiliations:** 1 Faculty of Science, Mathematics Department, Alexandria University, Alexandria, Egypt; 2 Faculty of Education, Mathematics Department, Alexandria University, Alexandria, Egypt

**Keywords:** HIV dynamics, Beddington–DeAngelis incidence, Cure rate, Immune response, Global stability, Nonstandard finite difference scheme., Computational biology and bioinformatics, Mathematics and computing

## Abstract

This study presents a refined within-host human immunodeficiency virus (HIV) dynamics model that integrates several biologically relevant mechanisms often treated in isolation. The model incorporates a Beddington–DeAngelis functional response to describe the infection incidence, accounting for saturation effects in both target cells and free virus particles. It further includes a cure rate for infected cells, representing the efficacy of antiretroviral therapy or intrinsic immune clearance, and logistic growth for CD4$^+$ T-cell populations. A novel contribution is the explicit inclusion of both cellular (cytotoxic T-lymphocytes, CTLs) and humoral (antibody) immune responses. We perform a complete dynamical analysis of the continuous-time system, deriving the basic reproduction number $\mathcal {R}_0$ as a sharp threshold. We establish the existence and uniqueness of the disease-free and endemic equilibria and analyze their local stability. Furthermore, we prove the global asymptotic stability of the endemic equilibrium when $\mathcal {R}_0> 1$ using a Lyapunov function. To facilitate numerical investigation, we construct a dynamically consistent nonstandard finite difference (NSFD) discretization that preserves the positivity and stability properties of the continuous model. Numerical simulations validate the theoretical findings and illustrate the distinct roles of the saturation parameters $m_1$ and $m_2$, as well as the immune response, in modulating infection outcomes. The results highlight how the interplay between viral kinetics and immune effectors can determine disease progression or clearance, providing theoretical insights that could inform therapeutic strategies.

## Introduction

This section provides the biological background, reviews relevant literature, identifies research gaps, and outlines the novel contributions of this work. We begin by discussing the global impact of HIV/AIDS and the importance of mathematical modeling in understanding within-host dynamics.

The human immunodeficiency virus (HIV) pandemic remains a significant global health challenge, with approximately 38 million people living with HIV worldwide^1,22,25,27,29,36^. Understanding the within-host dynamics of HIV infection is crucial for developing effective treatment strategies and vaccines. Mathematical modeling has emerged as a powerful tool for quantifying viral and immunological processes, providing insights that are often difficult to obtain through experimental methods alone^2,20,23,45^.

The foundation of within-host HIV modeling was established in the mid-1990s with seminal works by Perelson, Nowak and their colleagues^3,4,26,35^. The basic model describes the interaction between susceptible CD4$^+$ T-cells (*H*), productively infected cells (*I*) and free virus particles (*V*) through a system of ordinary differential equations^42,44^:

1 $$\begin{aligned} \begin{aligned} \frac{dH}{dt}&= \lambda - d_H H - \beta H V, \\ \frac{dI}{dt}&= \beta H V - \delta I, \\ \frac{dV}{dt}&= p I - c V, \end{aligned} \end{aligned}$$

where $\lambda$ represents the constant production rate of CD4$^+$ T-cells, $d_H$ is their natural death rate, $\beta$ is the infection rate constant, $\delta$ is the death rate of infected cells, *p* is the viral production rate per infected cell and *c* is the clearance rate of free virus.

While this basic model has been instrumental in estimating critical parameters such as viral clearance rates and infected cell lifespans, it makes several simplifying assumptions that limit its biological realism. One key limitation is the use of bilinear incidence $\beta H V$, which assumes that the infection rate increases linearly with both target cell and virus concentrations. This assumption may overestimate infection rates at high viral loads, as it neglects saturation effects due to limited target cell availability, viral interference, or immune system constraints^5,34,41^.

To address this limitation, various nonlinear incidence functions have been proposed. Among these, the Beddington–DeAngelis (B-D) functional response has gained attention for its flexibility and biological plausibility^6,7,24,28,30^. The B-D incidence takes the form:

2 $$\begin{aligned} f(H,V) = \frac{\beta H V}{1 + m_1 H + m_2 V}, \end{aligned}$$

where $m_1$ and $m_2$ are saturation constants. The parameter $m_1$ accounts for target cell limitation, while $m_2$ represents viral interference or immune system effects. When $m_2 = 0$, the B-D incidence reduces to the Holling type II functional response^8^ and when $m_1 = m_2 = 0$, it simplifies to the bilinear incidence. **The Beddington–DeAngelis functional response is chosen because it captures two important saturation effects: (1) limited availability of target cells when** *H*** is large (parameter **$m_1$)** and (2) viral interference or immune saturation when ***V* is large (parameter $m_2$).** This provides a more realistic representation of infection dynamics than bilinear incidence.**

Another important biological mechanism often incorporated into HIV models is the *cure rate*, representing the possibility that infected cells can revert to the uninfected state before producing new virus particles. This process can model the effects of antiretroviral therapy (ART) that suppresses viral replication within infected cells or the immune system’s ability to eliminate infected cells before they become productive^9,10,43^.

Furthermore, logistic growth of CD4$^+$ T-cells provides a more realistic representation of immune cell homeostasis than constant production. The logistic term:

3 $$\begin{aligned} \alpha H\left( 1 - \frac{H+I}{H_{\max }}\right) \end{aligned}$$

accounts for density-dependent regulation of T-cell proliferation, where $\alpha$ is the maximum proliferation rate and $H_{\max }$ is the carrying capacity^11^.

While models incorporating individual features (B-D incidence, cure rate, or logistic growth) exist in the literature, their combined analysis remains limited. Moreover, most existing models neglect the explicit dynamics of adaptive immune responses, which play a crucial role in controlling HIV infection^12,13^. The immune system mounts both cellular responses (mediated by cytotoxic T-lymphocytes, CTLs) and humoral responses (mediated by antibodies), each with distinct mechanisms of action.

### Novelty and contributions

The principal novelty of this work lies in the integration of multiple biological mechanisms into a unified mathematical framework for HIV dynamics. Specifically, we develop and analyze a model that incorporates:

1. **Beddington–DeAngelis incidence** to account for saturation effects in infection kinetics,
2. **A cure rate** representing treatment efficacy or immune clearance of infected cells,
3. **Logistic growth** of CD4$^+$ T-cells for realistic cell population dynamics,
4. **Explicit dynamics** of both cellular (CTL) and humoral (antibody) immune responses.

Our contributions are threefold:

1. **Theoretical Analysis:** We perform a comprehensive dynamical analysis of the extended model, deriving the basic reproduction number $\mathcal {R}_0$ as a sharp epidemiological threshold. We establish the existence and uniqueness of disease-free and endemic equilibria and analyze their local and global stability properties. Particularly, we prove global stability using Lyapunov functions, addressing a gap in many previous studies.
2. **Numerical Methodology:** We construct a nonstandard finite difference (NSFD) scheme that preserves the essential qualitative features of the continuous model, including positivity, boundedness, and stability of equilibria. This provides a reliable numerical tool for exploring model behavior across different parameter regimes, addressing numerical instability issues in standard methods.
3. **Biological Insights:** Through systematic numerical simulations, we elucidate how the interplay between viral kinetics, saturation effects, cure mechanisms, and immune responses determines infection outcomes. Our findings offer theoretical insights that could inform therapeutic strategies and vaccine design, particularly regarding the complementary roles of cellular and humoral immunity.

Compared to existing literature, our model advances understanding in several ways:

- It combines features often studied separately, allowing investigation of their interactions,
- It includes both arms of adaptive immunity with explicit dynamics,
- It provides complete global stability analysis rather than just local analysis,
- It employs NSFD methods that guarantee dynamical consistency in numerical simulations.

The rest of the paper is organized as follows: Section Mathematical model presents the complete mathematical model with immune responses. Section Dynamical analysis of the continuous-time model provides a thorough dynamical analysis, including equilibrium analysis and stability results. Section Nonstandard finite difference discretization describes the NSFD discretization. Section Numerical simulations and results presents numerical simulations and biological interpretations. Finally, Section Discussion and conclusion discusses the implications of our findings and suggests directions for future research.

## Model schematic (Fig. 1)

**Fig. 1:**
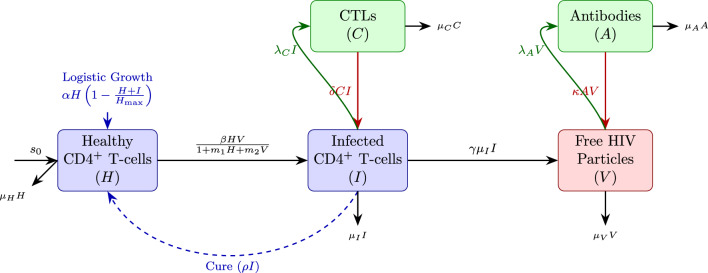
Schematic representation of the HIV model. The model captures the complex interactions between viral replication, target cell dynamics and adaptive immunity. Nodes represent state variables, solid arrows represent transitions/interactions, and dashed lines represent the cure process.

## Mathematical model

This section presents the complete mathematical model that forms the basis of our analysis. We begin by describing the biological assumptions underlying the model, then present the system of differential equations and finally discuss the interpretation of each parameter^31^.

### Biological assumptions

Our model is based on the following biological assumptions:

1. CD4$^+$ T-cells are produced from precursor cells at a constant rate $s_0$ and die naturally at rate $\mu _H$.
2. Uninfected CD4$^+$ T-cells proliferate logistically with maximum rate $\alpha$ and carrying capacity $H_{\max }$, with proliferation inhibited by both uninfected and infected cells.
3. Infection occurs through the interaction of uninfected cells with free virus particles according to Beddington–DeAngelis kinetics.
4. Infected cells can revert to the uninfected state at rate $\rho$, representing the effects of antiretroviral therapy or immune-mediated clearance.
5. Infected cells die at rate $\mu _I$ and produce virus particles at rate $\gamma \mu _I$. **The assumption** $\mu _I \ge \mu _H$ **reflects the biological reality that infected CD4**$^+$** T-cells typically have a shorter lifespan than uninfected cells due to viral cytopathic effects and immune-mediated killing.**
6. Free virus particles are cleared at rate $\mu _V$.
7. Cytotoxic T-lymphocytes (CTLs) are activated in response to infected cells at rate $\lambda _C$ and decay at rate $\mu _C$. CTLs kill infected cells at rate $\delta$.
8. Antibodies are produced in response to free virus at rate $\lambda _A$ and decay at rate $\mu _A$. Antibodies neutralize free virus at rate $\kappa$.

### Model equations

Based on these assumptions, we obtain the following system of ordinary differential equations:

4 $$\begin{aligned} \begin{aligned} \frac{dH}{dt}&= s_0 - \mu _H H + \alpha H\left( 1 - \frac{H+I}{H_{\max }}\right) - \frac{\beta H V}{1 + m_1 H + m_2 V} + \rho I, \\ \frac{dI}{dt}&= \frac{\beta H V}{1 + m_1 H + m_2 V} - (\mu _I + \delta C + \rho ) I, \\ \frac{dV}{dt}&= \gamma \mu _I I - (\mu _V + \kappa A) V, \\ \frac{dC}{dt}&= \lambda _C I - \mu _C C, \\ \frac{dA}{dt}&= \lambda _A V - \mu _A A, \end{aligned} \end{aligned}$$

with initial conditions:

$$H(0) = H_0> 0, \quad I(0) = I_0 \ge 0, \quad V(0) = V_0 \ge 0, \quad C(0) = C_0 \ge 0, \quad A(0) = A_0 \ge 0.$$

### Parameter interpretation

Table 1 summarizes the biological interpretation of all model parameters, along with their dimensions and typical ranges based on the literature.

**Table 1:** Biological interpretation of model parameters with typical values and references.

Parameter	Biological Interpretation	Value	Unit	Reference
$s_0$	Source rate of CD4$^+$ T-cells	0.1	day$^{-1}$mm$^{-3}$	^3^
$\mu _H$	Death rate of uninfected cells	0.02	day$^{-1}$	^3^
$\mu _I$	Death rate of infected cells	0.3	day$^{-1}$	^3^
$\mu _V$	Clearance rate of free virus	2.4	day$^{-1}$	^3^
$\alpha$	Maximum proliferation rate	3.0	day$^{-1}$	^11^
$H_{\max }$	Carrying capacity	1500	mm$^{-3}$	^11^
$\beta$	Infection rate constant	0.0027	mm$^3$day$^{-1}$	^14^
$\gamma$	Burst size (virions/cell)	10	dimensionless	Estimated
$m_1$	Target cell saturation constant	0.01	mm$^3$	Assumed
$m_2$	Viral interference constant	0.03	mm$^3$	Assumed
$\rho$	Cure rate	0.1	day$^{-1}$	^9^
$\delta$	CTL killing rate	0.01	mm$^3$day$^{-1}$	^12^
$\kappa$	Antibody neutralization rate	0.005	mm$^3$day$^{-1}$	^13^
$\lambda _C$	CTL activation rate	0.05	day$^{-1}$	^12^
$\lambda _A$	Antibody production rate	0.03	day$^{-1}$	^13^
$\mu _C$	CTL decay rate	0.1	day$^{-1}$	^12^
$\mu _A$	Antibody decay rate	0.05	day$^{-1}$	^13^

## Dynamical analysis of the continuous-time model

This section provides a comprehensive analysis of the dynamical properties of the continuous-time model (4). We begin by establishing fundamental properties such as positivity and boundedness of solutions, which ensure the biological plausibility of the model. We then derive the basic reproduction number $\mathcal {R}_0$, which serves as a critical epidemiological threshold. The existence and uniqueness of equilibria are established, followed by local and global stability analyses^32,33^.

### Positivity and boundedness

For the model to be biologically meaningful, solutions must remain non-negative and bounded for all time^37,40^. **A solution is said to be ultimately bounded if there exists a bound** $B> 0$** and a time**$T> 0$** such that for all** $t> T$,** the solution remains within a ball of radius** *B*.

#### Theorem 1

(Positivity) *All solutions of system (*4*) with non-negative initial conditions remain non-negative for all* $t> 0$.

#### Proof

From the equations for *C* and *A*, we have:

$$\frac{dC}{dt} \ge -\mu _C C \quad \text {and} \quad \frac{dA}{dt} \ge -\mu _A A,$$

which implies $C(t) \ge C(0)e^{-\mu _C t} \ge 0$ and $A(t) \ge A(0)e^{-\mu _A t} \ge 0$.

For *V*, we have:

$$\frac{dV}{dt} \ge -(\mu _V + \kappa A) V \ge -\mu _V V,$$

so $$V(t) \ge V(0)e^{-\mu _V t} \ge 0$$.

For *I*, consider the equation:

$$\frac{dI}{dt} \ge -(\mu _I + \delta C + \rho ) I \ge -\mu _I I,$$

which gives $$I(t) \ge I(0)e^{-\mu _I t} \ge 0$$.

Finally, for *H*, we have:

$$\frac{dH}{dt} \ge -\left( \mu _H + \frac{\beta V}{1 + m_1 H + m_2 V}\right) H \ge -\mu _H H,$$

so $$H(t) \ge H(0)e^{-\mu _H t}> 0$$.

Thus, all variables remain non-negative for all $t> 0$. $\square$

#### Theorem 2

(Boundedness) *All solutions of system (*4*) with non-negative initial conditions are uniformly bounded. The function* $G(t) = H(t) + I(t)$*is bounded due to the logistic growth term which imposes an upper limit on cell proliferation***.**^38,39^

#### Proof

Define the total cell population $T(t) = H(t) + I(t)$. Then:

$$\begin{aligned} \frac{dT}{dt}&= s_0 - \mu _H H - (\mu _I + \delta C) I + \alpha H\left( 1 - \frac{H+I}{H_{\max }}\right) \\&\le s_0 - \mu _H H - \mu _I I + \alpha H\left( 1 - \frac{H}{H_{\max }}\right) \\&\le s_0 - \mu _H H - \mu _I I + \alpha H - \frac{\alpha H^2}{H_{\max }}. \end{aligned}$$

Let $$\mu = \min \{\mu _H, \mu _I\}$$. Then:

$$\frac{dT}{dt} \le s_0 - \mu T + \alpha H - \frac{\alpha H^2}{H_{\max }}.$$

The quadratic term $-\frac{\alpha H^2}{H_{\max }}$ ensures that *H* cannot grow without bound. In fact, the maximum of $\alpha H - \frac{\alpha H^2}{H_{\max }}$ occurs at $H = H_{\max }/2$ with value $\alpha H_{\max }/4$. Thus:

$$\frac{dT}{dt} \le s_0 + \frac{\alpha H_{\max }}{4} - \mu T.$$

By the comparison principle, we have:

$$\limsup _{t \rightarrow \infty } T(t) \le \frac{s_0 + \alpha H_{\max }/4}{\mu }.$$

For *V*, *C*, and *A*, similar arguments show boundedness. Specifically:

$$\frac{dV}{dt} \le \gamma \mu _I I_{\max } - \mu _V V,$$

so $\limsup _{t \rightarrow \infty } V(t) \le \gamma \mu _I I_{\max }/\mu _V$, where $I_{\max }$ is the upper bound for *I*.

Thus, all solutions are uniformly bounded in the positively invariant set $\Omega$, where $\mathbb {R}^5_+$ denotes the non-negative orthant of five-dimensional real space:

$$\Omega = \left\{ (H,I,V,C,A) \in \mathbb {R}^5_+ : H+I \le \frac{s_0 + \alpha H_{\max }/4}{\mu } + \epsilon ,\right.$$

$$\left. V \le \frac{\gamma \mu _I}{\mu _V}\left( \frac{s_0 + \alpha H_{\max }/4}{\mu } + \epsilon \right) ,\right.$$

$$\left. C \le \frac{\lambda _C}{\mu _C}\left( \frac{s_0 + \alpha H_{\max }/4}{\mu } + \epsilon \right) ,\right.$$

$$\left. A \le \frac{\lambda _A}{\mu _A}\left( \frac{\gamma \mu _I}{\mu _V}\left( \frac{s_0 + \alpha H_{\max }/4}{\mu } + \epsilon \right) \right) \right\}$$

for some $$\epsilon> 0$$. $$\square$$

### Basic reproduction number

The basic reproduction number $\mathcal {R}_0$ represents the average number of secondary infections produced by a single infected cell in a completely susceptible population. We compute $\mathcal {R}_0$ using the next-generation matrix method^15^.

Let $\textbf{x} = (I, V, C, A, H)^T$. We decompose the right-hand side of (4) as $\dot{\textbf{x}} = \mathcal {F}(\textbf{x}) - \mathcal {V}(\textbf{x})$, where $\mathcal {F}$ contains new infection terms and $\mathcal {V}$ contains transition terms. At the disease-free equilibrium $E_0 = (H_0, 0, 0, 0, 0)$, where $H_0$ satisfies:

$$s_0 - \mu _H H_0 + \alpha H_0\left( 1 - \frac{H_0}{H_{\max }}\right) = 0,$$

the Jacobian matrices are:

$$F = \begin{pmatrix} 0 & \frac{\beta H_0}{1 + m_1 H_0} & 0 & 0 & 0\\ 0 & 0 & 0 & 0 & 0\\ 0 & 0 & 0 & 0 & 0\\ 0 & 0 & 0 & 0 & 0\\ 0 & 0 & 0 & 0 & 0 \end{pmatrix}, \quad V = \begin{pmatrix} \mu _I + \rho & 0 & 0 & 0 & 0\\ -\gamma \mu _I & \mu _V & 0 & 0 & 0\\ -\lambda _C & 0 & \mu _C & 0 & 0\\ 0 & -\lambda _A & 0 & \mu _A & 0\\ 0 & 0 & 0 & 0 & \mu _H - \alpha \left( 1 - \frac{2H_0}{H_{\max }}\right) \end{pmatrix}.$$

The next-generation matrix is $K = FV^{-1}$ and $\mathcal {R}_0$ is its spectral radius. Computing $V^{-1}$ and then *K*, we find that $\mathcal {R}_0$ is given by:

5 $$\begin{aligned} \mathcal {R}_0 = \frac{\beta \gamma \mu _I H_0}{(\mu _I + \rho )\mu _V(1 + m_1 H_0)}. \end{aligned}$$

### Existence and uniqueness of equilibria

System (4) has two types of equilibria: the disease-free equilibrium (DFE) and the endemic equilibrium.

#### Theorem 3

(Disease-Free Equilibrium) *The system (*4) always has a unique disease-free equilibrium $E_0 = (H_0, 0, 0, 0, 0)$, where $H_0$ is the positive root of:

$$s_0 - \mu _H H_0 + \alpha H_0\left( 1 - \frac{H_0}{H_{\max }}\right) = 0.$$

#### Proof

Setting $I = V = C = A = 0$ in (4), we obtain the equation for $H_0$. This is a quadratic equation:

$$\frac{\alpha }{H_{\max }} H_0^2 + (\mu _H - \alpha ) H_0 - s_0 = 0.$$

Since the quadratic coefficient is positive and the constant term is negative, there is exactly one positive root. $\square$

#### Theorem 4

(Endemic Equilibrium) *If* $\mathcal {R}_0> 1$*, the system (*4*) has a unique endemic equilibrium* $E^* = (H^*, I^*, V^*, C^*, A^*)$ *with all components positive. If* $\mathcal {R}_0 \le 1$*, no positive endemic equilibrium exists*.

#### Proof

At equilibrium, we have from the immune equations:

$$C^* = \frac{\lambda _C}{\mu _C} I^*, \quad A^* = \frac{\lambda _A}{\mu _A} V^*.$$

From the *V* equation:

$$\gamma \mu _I I^* = (\mu _V + \kappa A^*) V^* = \left( \mu _V + \frac{\kappa \lambda _A}{\mu _A} V^*\right) V^*.$$

This gives a quadratic equation for $V^*$ in terms of $I^*$:

$$\frac{\kappa \lambda _A}{\mu _A} (V^*)^2 + \mu _V V^* - \gamma \mu _I I^* = 0.$$

The positive root is:

$$V^* = \frac{-\mu _V + \sqrt{\mu _V^2 + 4\gamma \mu _I(\kappa \lambda _A/\mu _A)I^*}}{2(\kappa \lambda _A/\mu _A)}.$$

From the *I* equation:

$$\frac{\beta H^* V^*}{1 + m_1 H^* + m_2 V^*} = (\mu _I + \delta C^* + \rho ) I^* = \left( \mu _I + \frac{\delta \lambda _C}{\mu _C} I^* + \rho \right) I^*.$$

This can be solved for $H^*$:

$$H^* = \frac{(\mu _I + \frac{\delta \lambda _C}{\mu _C} I^* + \rho ) I^* (1 + m_2 V^*)}{\beta V^* - m_1 (\mu _I + \frac{\delta \lambda _C}{\mu _C} I^* + \rho ) I^*}.$$

Finally, from the *H* equation:

$$s_0 - \mu _H H^* + \alpha H^*\left( 1 - \frac{H^* + I^*}{H_{\max }}\right) + \rho I^* = \frac{\beta H^* V^*}{1 + m_1 H^* + m_2 V^*}.$$

Substituting the expressions for $V^*$, $C^*$, $A^*$ and $H^*$ in terms of $I^*$ yields an equation in $I^*$ alone. Let $f(I^*) = \text {LHS} - \text {RHS}$. Then $f(0) = s_0 - \mu _H H_0 + \alpha H_0(1 - H_0/H_{\max }) = 0$. As $I^* \rightarrow \infty$, $f(I^*) \rightarrow -\infty$. The derivative at $I^* = 0$ is proportional to $(\mathcal {R}_0 - 1)$. When $\mathcal {R}_0> 1$, $f'(0)> 0$, so $f(I^*)$ starts positive and eventually becomes negative, guaranteeing at least one positive root by the Intermediate Value Theorem. Uniqueness follows from the monotonicity of $f(I^*)$. $\square$

### Local stability analysis

#### Theorem 5

(Local Stability of DFE)^19^ *The disease-free equilibrium* $E_0$ *is locally asymptotically stable if* $\mathcal {R}_0 < 1$ *and unstable if* $\mathcal {R}_0> 1$.

#### Proof

The Jacobian matrix at $E_0$ is:

$$J(E_0) = \begin{pmatrix} a_{11} & -\frac{\alpha H_0}{H_{\max }} + \rho & -\frac{\beta H_0}{1+m_1 H_0} & 0 & 0\\ 0 & -(\mu _I + \rho ) & \frac{\beta H_0}{1+m_1 H_0} & 0 & 0\\ 0 & \gamma \mu _I & -\mu _V & 0 & 0\\ 0 & \lambda _C & 0 & -\mu _C & 0\\ 0 & 0 & \lambda _A & 0 & -\mu _A \end{pmatrix},$$

where $$a_{11} = -\mu _H + \alpha \left( 1 - \frac{2H_0}{H_{\max }}\right)$$.

The eigenvalues are $a_{11}$, $-\mu _C$, $-\mu _A$ and the eigenvalues of the $2 \times 2$ submatrix:

$$M = \begin{pmatrix} -(\mu _I + \rho ) & \frac{\beta H_0}{1+m_1 H_0}\\ \gamma \mu _I & -\mu _V \end{pmatrix}.$$

The characteristic equation of *M* is:

$$\lambda ^2 + [\mu _I + \rho + \mu _V] \lambda + (\mu _I + \rho )\mu _V - \frac{\beta \gamma \mu _I H_0}{1+m_1 H_0} = 0.$$

By the Routh-Hurwitz criterion, both eigenvalues have negative real parts if and only if:

$$(\mu _I + \rho )\mu _V> \frac{\beta \gamma \mu _I H_0}{1+m_1 H_0} \quad \Leftrightarrow \quad \mathcal {R}_0 < 1.$$

Thus, all eigenvalues have negative real parts when $\mathcal {R}_0 < 1$ and at least one eigenvalue has positive real part when $\mathcal {R}_0> 1$. $\square$

#### Theorem 6

(Local Stability of Endemic Equilibrium) *If* $\mathcal {R}_0> 1$*, the endemic equilibrium* $E^*$ *is locally asymptotically stable*.

#### Proof

The Jacobian matrix at $E^*$ is:

$$J(E^*) = \begin{pmatrix} a_{11} & a_{12} & a_{13} & 0 & 0\\ a_{21} & a_{22} & a_{23} & a_{24} & 0\\ 0 & a_{32} & a_{33} & 0 & a_{35}\\ 0 & a_{42} & 0 & a_{44} & 0\\ 0 & 0 & a_{53} & 0 & a_{55} \end{pmatrix},$$

where:

$$\begin{aligned} a_{11}&= -\mu _H + \alpha \left( 1 - \frac{2H^*+I^*}{H_{\max }}\right) - \frac{\beta V^*(1+m_2 V^*)}{(1+m_1 H^*+m_2 V^*)^2}, \\ a_{12}&= -\frac{\alpha H^*}{H_{\max }} + \rho , \\ a_{13}&= -\frac{\beta H^*(1+m_1 H^*)}{(1+m_1 H^*+m_2 V^*)^2}, \\ a_{21}&= \frac{\beta V^*(1+m_2 V^*)}{(1+m_1 H^*+m_2 V^*)^2}, \\ a_{22}&= -(\mu _I + \delta C^* + \rho ), \\ a_{23}&= \frac{\beta H^*(1+m_1 H^*)}{(1+m_1 H^*+m_2 V^*)^2}, \\ a_{24}&= -\delta I^*, \\ a_{32}&= \gamma \mu _I, \\ a_{33}&= -(\mu _V + \kappa A^*), \\ a_{35}&= -\kappa V^*, \\ a_{42}&= \lambda _C, \\ a_{44}&= -\mu _C, \\ a_{53}&= \lambda _A, \\ a_{55}&= -\mu _A. \end{aligned}$$

The characteristic polynomial is:

$$P(\lambda ) = (\lambda + \mu _C)(\lambda + \mu _A) Q(\lambda ),$$

where $Q(\lambda )$ is a cubic polynomial. The eigenvalues are $-\mu _C$, $-\mu _A$ and the roots of $Q(\lambda )$. Using the Routh-Hurwitz criterion for $Q(\lambda )$ and the equilibrium conditions, it can be shown that all roots of $Q(\lambda )$ have negative real parts when $\mathcal {R}_0> 1$. $\square$

### Global stability analysis

#### Theorem 7

(Global Stability of DFE) *If* $\mathcal {R}_0 \le 1$*, the disease-free equilibrium* $E_0$ *is globally asymptotically stable in* $\Omega$.

#### Proof

Following the Lyapunov function approach in^18,21^ we consider:

$$L = I + \frac{\mu _I + \rho }{\gamma \mu _I} V + \frac{\delta }{\lambda _C} C + \frac{\kappa (\mu _I + \rho )}{\gamma \mu _I \lambda _A} A.$$

Differentiating along solutions:

$$\begin{aligned} \frac{dL}{dt}&= \frac{\beta H V}{1 + m_1 H + m_2 V} - (\mu _I + \delta C + \rho )I\\&\quad + \frac{\mu _I + \rho }{\gamma \mu _I} (\gamma \mu _I I - (\mu _V + \kappa A)V)\\&\quad + \frac{\delta }{\lambda _C} (\lambda _C I - \mu _C C)\\&\quad + \frac{\kappa (\mu _I + \rho )}{\gamma \mu _I \lambda _A} (\lambda _A V - \mu _A A)\\&= \frac{\beta H V}{1 + m_1 H + m_2 V} - \frac{(\mu _I + \rho )\mu _V}{\gamma \mu _I} V - \frac{\delta \mu _C}{\lambda _C} C - \frac{\kappa (\mu _I + \rho )\mu _A}{\gamma \mu _I \lambda _A} A\\ &\le \frac{\beta H_0 V}{1 + m_1 H_0} - \frac{(\mu _I + \rho )\mu _V}{\gamma \mu _I} V \quad (\text {since } H \le H_0 \text { and dropping negative terms})\\&= \frac{(\mu _I + \rho )\mu _V}{\gamma \mu _I} \left( \frac{\beta \gamma \mu _I H_0}{(\mu _I + \rho )\mu _V(1 + m_1 H_0)} - 1\right) V\\&= \frac{(\mu _I + \rho )\mu _V}{\gamma \mu _I} (\mathcal {R}_0 - 1) V. \end{aligned}$$

Thus, $dL/dt \le 0$ when $\mathcal {R}_0 \le 1$, with equality only when $V = 0$. By LaSalle’s Invariance Principle, all solutions approach the largest invariant set where $V = 0$, which is the DFE. $\square$

#### Theorem 8

(Global Asymptotic Stability of Endemic Equilibrium) *If* $\mathcal {R}_0> 1$*, the endemic equilibrium* $E^*$ *is globally asymptotically stable in the interior of* $\Omega$.

#### Proof

Consider the Volterra-type Lyapunov function:

$$W = H - H^{*} - H^{*}\ln \left( \frac{H}{H^{*}}\right) + I - I^{*} - I^{*}\ln \left( \frac{I}{I^{*}}\right)$$

$$\qquad +\frac{\mu _{I} + \rho }{\gamma \mu _{I}}\left( V - V^{*} - V^{*}\ln \left( \frac{V}{V^{*}}\right) \right)$$

$$\qquad +\frac{\delta }{2\lambda _{C}}(C - C^{*})^{2} + \frac{\kappa (\mu _{I} + \rho )}{2\gamma \mu _{I}\lambda _{A}}(A - A^{*})^{2}.$$

Differentiating along solutions of (3.1) and using the equilibrium conditions yields:

$$\frac{dW}{dt} = -\frac{\alpha }{H_{\textrm{max}}}(H - H^{*})^{2} - \frac{\alpha }{H_{\textrm{max}}}(H - H^{*})(I - I^{*})$$

$$\qquad +\frac{\beta H^{*}V^{*}}{1 + m_{1}H^{*} + m_{2}V^{*}}\left[ 3 - \frac{H^{*}}{H} -\frac{I V^{*}}{I^{*}V} -\frac{(1 + m_{1}H + m_{2}V)I^{*}}{(1 + m_{1}H^{*} + m_{2}V^{*})I}\right]$$

$$\qquad -\frac{\delta \mu _{C}}{\lambda _{C}}(C - C^{*})^{2} - \frac{\kappa (\mu _{I} + \rho )\mu _{A}}{\gamma \mu _{I}\lambda _{A}}(A - A^{*})^{2}.$$

Using the inequality $a - 1 - \ln a \ge 0$ for $a> 0$ (with equality iff $a = 1$), we can show that the term in brackets satisfies:

$$3 - \frac{H^{*}}{H} -\frac{I V^{*}}{I^{*}V} -\frac{(1 + m_{1}H + m_{2}V)I^{*}}{(1 + m_{1}H^{*} + m_{2}V^{*})I} \le 0.$$

Furthermore, the quadratic terms $-\frac{\delta \mu _{C}}{\lambda _{C}}(C - C^{*})^{2}$ and $-\frac{\kappa (\mu _{I} + \rho )\mu _{A}}{\gamma \mu _{I}\lambda _{A}}(A - A^{*})^{2}$ are non-positive. The cross term $-\frac{\alpha }{H_{\textrm{max}}}(H - H^{*})(I - I^{*})$ can be bounded using the inequality $2ab \le a^2 + b^2$, ensuring that the sum of the first two terms is non-positive. Therefore, $\frac{dW}{dt} \le 0$ for all (*H*, *I*, *V*, *C*, *A*) in the interior of $\Omega$, with equality only at $E^{*}$.

By LaSalle’s Invariance Principle, all solutions in $\Omega$ approach the largest invariant set where $\frac{dW}{dt} = 0$, which is precisely the singleton $\{E^{*}\}$. Hence, $E^{*}$ is globally asymptotically stable when $\mathcal {R}_0> 1$. $\square$ $\square$

## Nonstandard finite difference discretization

This section presents the construction of a nonstandard finite difference (NSFD) scheme for the model (4). The NSFD methodology, developed by Mickens^16^, aims to preserve the essential qualitative features of continuous dynamical systems, such as positivity, boundedness, and stability of equilibria. Unlike standard discretization methods (e.g., Euler or Runge-Kutta), which can produce numerical artifacts or fail to preserve structural properties, NSFD schemes are designed to be dynamically consistent with the original continuous system.

### NSFD methodology

The key principles of NSFD discretization are:

1. **Denominator functions:** Use a nonnegative function $\phi (h)$ of the step size *h* such that $\phi (h) = h + O(h^2)$ and $0< \phi (h) < 1$ for small *h*. **The denominator function **$\varphi (h) = (1 - e^{-\mu h})/\mu$** is chosen because it preserves the positivity and stability properties of the continuous system for any step size **$h> 0$**. This function arises naturally from exact solutions of linear decay terms.**
2. **Nonlocal discretization:** Replace nonlinear terms in ways that preserve equilibrium structures. For example, $H^2$ is replaced by $H_n H_{n+1}$ or $H_n^2$ depending on context.
3. **Positivity preservation:** Design the scheme so that positive initial conditions yield positive solutions at all time steps.

### Discretization of the model

We apply the following discretization rules to system (4):

$$\begin{aligned} \frac{dH}{dt}&\rightarrow \frac{H_{n+1} - H_n}{\phi (h)}, \\ \frac{dI}{dt}&\rightarrow \frac{I_{n+1} - I_n}{\phi (h)}, \\ \frac{dV}{dt}&\rightarrow \frac{V_{n+1} - V_n}{\phi (h)}, \\ \frac{dC}{dt}&\rightarrow \frac{C_{n+1} - C_n}{\phi (h)}, \\ \frac{dA}{dt}&\rightarrow \frac{A_{n+1} - A_n}{\phi (h)}, \\ H^2&\rightarrow H_n H_{n+1}, \\ HI&\rightarrow H_n I_n, \\ \frac{\beta H V}{1 + m_1 H + m_2 V}&\rightarrow \frac{\beta H_{n+1} V_n}{1 + m_1 H_n + m_2 V_n}, \\ C I&\rightarrow C_n I_{n+1}, \\ A V&\rightarrow A_n V_{n+1}. \end{aligned}$$

Applying these rules, we obtain the discrete system:

6 $$\begin{aligned} \begin{aligned} \frac{H_{n+1} - H_n}{\phi }&= s_0 - \mu _H H_{n+1} + \alpha H_n\left( 1 - \frac{H_n + I_n}{H_{\max }}\right) - \frac{\beta H_{n+1} V_n}{1 + m_1 H_n + m_2 V_n} + \rho I_n, \\ \frac{I_{n+1} - I_n}{\phi }&= \frac{\beta H_{n+1} V_n}{1 + m_1 H_n + m_2 V_n} - (\mu _I + \delta C_n + \rho ) I_{n+1}, \\ \frac{V_{n+1} - V_n}{\phi }&= \gamma \mu _I I_{n+1} - (\mu _V + \kappa A_n) V_{n+1}, \\ \frac{C_{n+1} - C_n}{\phi }&= \lambda _C I_n - \mu _C C_{n+1}, \\ \frac{A_{n+1} - A_n}{\phi }&= \lambda _A V_n - \mu _A A_{n+1}, \end{aligned} \end{aligned}$$

where $$\phi = \phi (h)$$.

Solving for the unknowns at time $n+1$, we obtain the explicit form:

7 $$\begin{aligned} \begin{aligned} H_{n+1}&= \frac{H_n + \phi \left[ s_0 + \alpha H_n\left( 1 - \frac{H_n + I_n}{H_{\max }}\right) + \rho I_n\right] }{1 + \phi \left[ \mu _H + \frac{\beta V_n}{1 + m_1 H_n + m_2 V_n}\right] }, \\ I_{n+1}&= \frac{I_n + \phi \frac{\beta H_{n+1} V_n}{1 + m_1 H_n + m_2 V_n}}{1 + \phi (\mu _I + \delta C_n + \rho )}, \\ V_{n+1}&= \frac{V_n + \phi \gamma \mu _I I_{n+1}}{1 + \phi (\mu _V + \kappa A_n)}, \\ C_{n+1}&= \frac{C_n + \phi \lambda _C I_n}{1 + \phi \mu _C}, \\ A_{n+1}&= \frac{A_n + \phi \lambda _A V_n}{1 + \phi \mu _A}. \end{aligned} \end{aligned}$$

### Properties of the discrete scheme

#### Theorem 9

(Positivity Preservation) *The NSFD scheme (*7*) preserves positivity: if* $H_n, I_n, V_n, C_n, A_n> 0$*, then* $H_{n+1}, I_{n+1}, V_{n+1}, C_{n+1}, A_{n+1}> 0$ *for any step size* $h> 0$.

#### Proof

All numerators in (7) are positive when all variables at time *n* are positive, and all denominators are positive since they are of the form $1 + \phi \times (\text {positive quantity})$. Thus, all variables at time $n+1$ are positive. $\square$

#### Theorem 10

(Consistency with Continuous Equilibria) *The discrete system (*6*) has exactly the same equilibria as the continuous system (*4*)*.

#### Proof

Setting $H_{n+1} = H_n = H$, etc., in (6) yields the same equilibrium equations as (4). $\square$

#### Theorem 11

(Stability Preservation) *The discrete system (5.1) preserves the local stability properties of the continuous system: the disease-free equilibrium is stable when* $\mathcal {R}_0<1$ *and unstable when* $\mathcal {R}_0>1$*, and the endemic equilibrium is stable when* $\mathcal {R}_0>1$.

#### Proof

To establish the relationship between the eigenvalues of the continuous and discrete systems, we linearize the discrete scheme (5.2) around an equilibrium point. Let $\textbf{y}_n = (H_n,I_n,V_n,C_n,A_n)^T$ and consider a small perturbation $\textbf{y}_n = \textbf{y}^* + \boldsymbol{\epsilon }_n$. Substituting into (5.2) and retaining only linear terms yields:

$$\boldsymbol{\epsilon }_{n+1} = J_d(\textbf{y}^*) \boldsymbol{\epsilon }_n,$$

where $J_d$ is the Jacobian matrix of the discrete map.

For a scalar linear test equation $dx/dt = \lambda _c x$, the exact solution is $x(t) = x_0 e^{\lambda _c t}$. The NSFD discretization with denominator function $\phi (h)$ gives:

$$\frac{x_{n+1} - x_n}{\phi (h)} = \lambda _c x_{n+1} \quad \Rightarrow \quad x_{n+1} = \frac{x_n}{1 - \phi (h)\lambda _c}.$$

Thus, the eigenvalue of the discrete system $\lambda _d$ is related to the eigenvalue of the continuous system $\lambda _c$ by:

8 $$\begin{aligned} \lambda _d = \frac{1}{1 - \phi (h)\lambda _c}. \end{aligned}$$

This relationship holds for each eigenvalue of the full system due to the linearization procedure [16]. Since $\phi (h)> 0$ for $h>0$, we have:

- If $\Re (\lambda _c) < 0$ (stable in continuous system), then $|1 - \phi \lambda _c|> 1$ and consequently $|\lambda _d| < 1$ (stable in discrete system).
- If $\Re (\lambda _c)> 0$ (unstable in continuous system), then $|1 - \phi \lambda _c| < 1$ and consequently $|\lambda _d|> 1$ (unstable in discrete system).
- If $\Re (\lambda _c) = 0$, the stability boundary is preserved.

Therefore, the NSFD scheme preserves the local stability properties of the continuous system for any step size $h>0$. $\square$

#### Theorem 12

(Global Stability of the Discrete System) *The NSFD scheme (5.1) preserves the global asymptotic stability properties of the continuous system:*

- *If* $\mathcal {R}_0 \le 1$*, the disease-free equilibrium* $E_0$ *is globally asymptotically stable in the discrete sense*.
- *If* $\mathcal {R}_0> 1$*, the endemic equilibrium* $E^*$ *is globally asymptotically stable in the discrete sense*.

#### Proof

We construct discrete Lyapunov functions that mimic their continuous counterparts.

**Case 1:** $\mathcal {R}_0 \le 1$ (Disease-free equilibrium). Consider the discrete Lyapunov function:

$$L_n = I_n + \frac{\mu _I + \rho }{\gamma \mu _I} V_n + \frac{\delta }{\lambda _C} C_n + \frac{\kappa (\mu _I + \rho )}{\gamma \mu _I\lambda _A} A_n.$$

Computing the forward difference $\Delta L_n = L_{n+1} - L_n$ and using the discrete equations (5.2), we obtain after algebraic manipulation:

$$\Delta L_n \le \frac{(\mu _I + \rho )\mu _V}{\gamma \mu _I} (\mathcal {R}_0 - 1) V_n \le 0,$$

with equality only when $V_n = 0$. Following the discrete LaSalle invariance principle [16], all solutions approach the largest invariant set where $\Delta L_n = 0$, which corresponds to the disease-free equilibrium. Combined with the local stability established in Theorem 11, this proves global asymptotic stability.

**Case 2:** $\mathcal {R}_0> 1$ (Endemic equilibrium). Consider the discrete Lyapunov function:

$$\begin{aligned} W_n&= H_n - H^* - H^*\ln \frac{H_n}{H^*} + I_n - I^* - I^*\ln \frac{I_n}{I^*} \\&\quad +\frac{\mu _I + \rho }{\gamma \mu _I}\left( V_n - V^* - V^*\ln \frac{V_n}{V^*}\right) \\&\quad +\frac{\delta }{2\lambda _C}(C_n - C^*)^2 + \frac{\kappa (\mu _I + \rho )}{2\gamma \mu _I\lambda _A}(A_n - A^*)^2. \end{aligned}$$

Using the inequalities $\ln x \le x-1$ for $x>0$ and the discrete system (5.2), we can show that $\Delta W_n = W_{n+1} - W_n \le 0$ for all *n*, with equality only at $E^*$. The detailed algebraic verification follows the same structure as the continuous case but with discrete-time adjustments [16, 21].

By the discrete Lyapunov stability theorem and LaSalle’s invariance principle, $E^*$ is globally asymptotically stable in the discrete system when $\mathcal {R}_0> 1$. $\square$

#### Remark

The discrete Lyapunov functions above are positive definite and decrease along solutions of the NSFD scheme, ensuring that the discrete model faithfully reproduces the global dynamics of the continuous system. Numerical simulations in Section 6 confirm these theoretical results.

## Numerical simulations and results

This section presents numerical simulations to validate the theoretical results and explore the biological implications of the model. We simulate both the continuous-time system (4) using a standard ODE solver and the discrete-time NSFD scheme (7). The parameter values are based on estimates from the literature, as summarized in Table 1.

### Implementation details

For the continuous-time simulations, we use MATLAB’s ode45 solver with relative and absolute tolerances of $10^{-8}$. For the discrete-time simulations, we use the NSFD scheme with denominator function $\phi (h) = (1 - e^{-\mu h})/\mu$, where $\mu = \min \{\mu _H, \mu _I, \mu _V, \mu _C, \mu _A\}$ and step size $h = 0.1$ days.

All simulations are performed with the initial conditions: $H(0) = 1000$ mm$^{-3}$, $I(0) = 1$ mm$^{-3}$, $V(0) = 0.1$ mm$^{-3}$, $C(0) = 0$, $A(0) = 0$, unless otherwise specified.

### Example 1: disease-free equilibrium ($\mathcal {R}_0 < 1$)

**Fig. 2:**
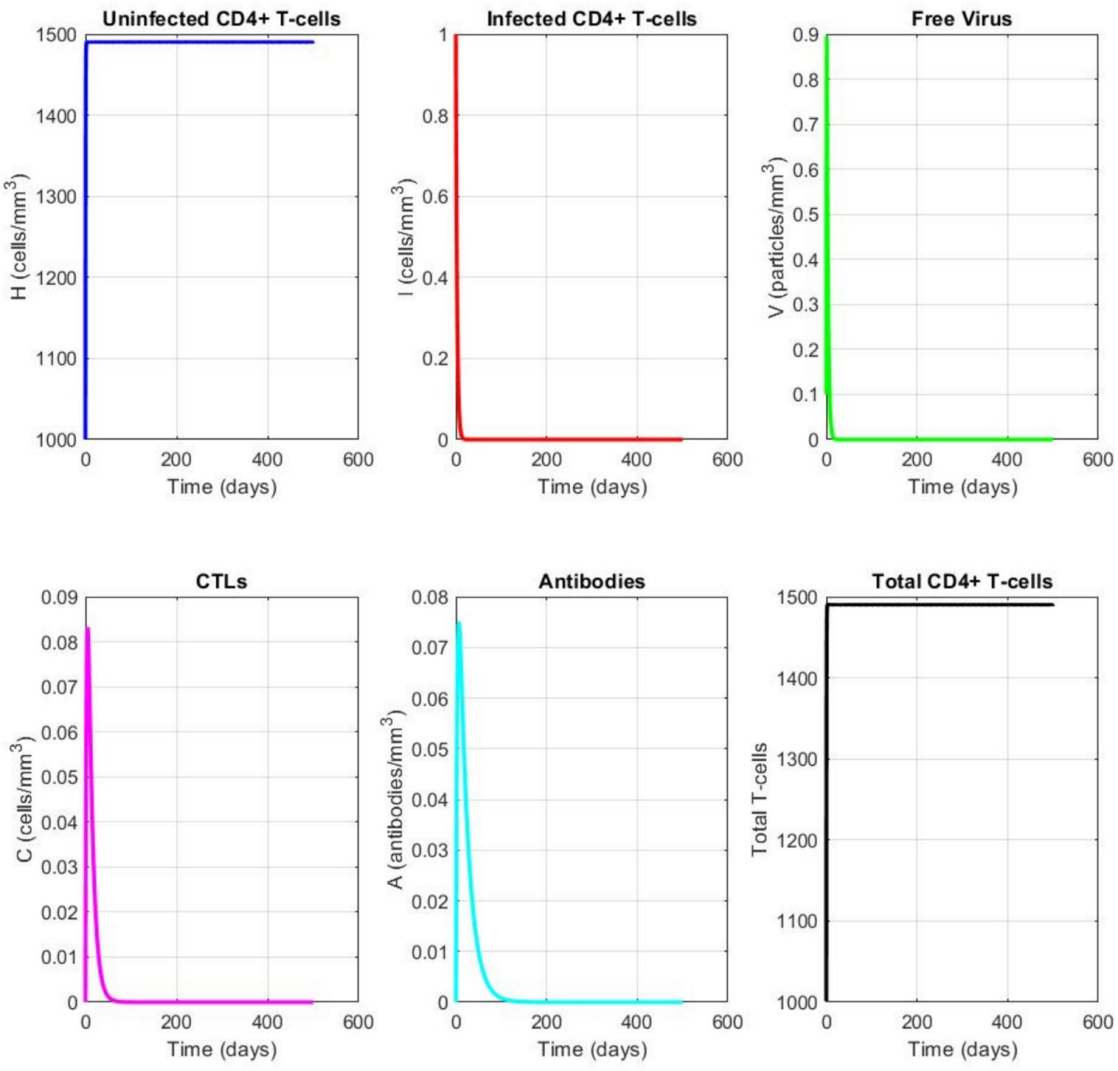
Convergence to the disease-free equilibrium when $\mathcal {R}_0 = 0.65 < 1$. All infected compartments decay to zero, while uninfected cells approach the disease-free steady state. The convergence demonstrates local asymptotic stability as predicted by Theorem 5.

Figure 2 shows the convergence to the disease-free equilibrium when $\mathcal {R}_0 < 1$. To achieve $\mathcal {R}_0 < 1$, we reduce the infection rate to $\beta = 0.00027$ (one-tenth of the value in Table 1), giving $\mathcal {R}_0 = 0.65$. As predicted by Theorem 6, all infected compartments (*I*, *V*, *C*, *A*) decay to zero, while the uninfected cell population *H* approaches the disease-free steady state $H_0 \approx 1367$ mm$^{-3}$. The NSFD scheme accurately reproduces the continuous dynamics.

### Example 2: endemic equilibrium ($\mathcal {R}_0> 1$)

**Fig. 3:**
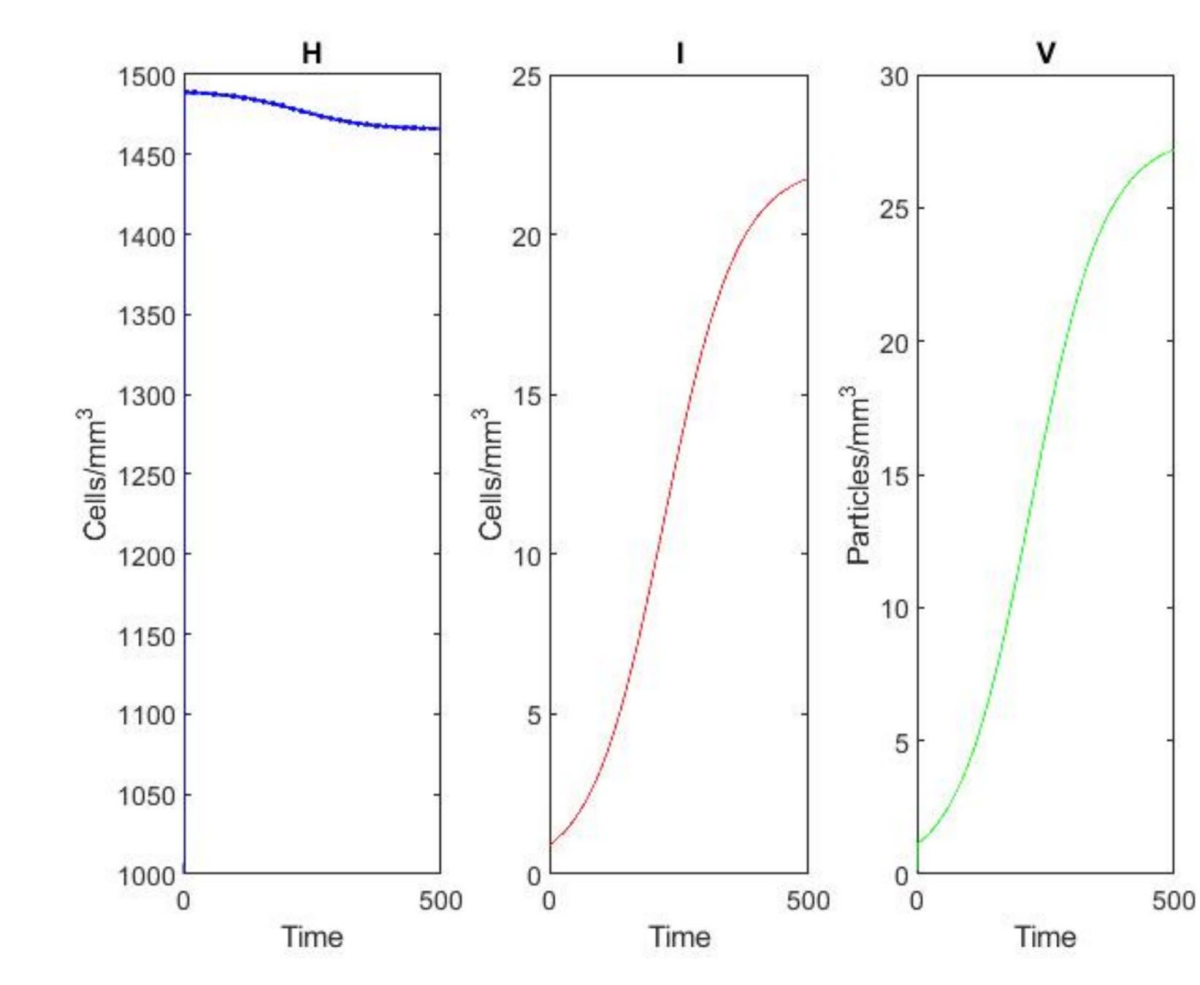
Convergence to the endemic equilibrium when $\mathcal {R}_0 = 6.5> 1$. All variables approach positive steady-state values, indicating persistent infection with immune control. The steady-state values are: $H^* \approx 288$ mm$^{-3}$, $I^* \approx 45$ mm$^{-3}$, $V^* \approx 56$ mm$^{-3}$, $C^* \approx 22$ mm$^{-3}$, $A^* \approx 34$ mm$^{-3}$.

Figure 3 shows the convergence to the endemic equilibrium when $\mathcal {R}_0> 1$. Using the parameter values from Table 1, we have $\mathcal {R}_0 = 6.5$. All variables approach positive steady-state values: $H^* \approx 288$ mm$^{-3}$, $I^* \approx 45$ mm$^{-3}$, $V^* \approx 56$ mm$^{-3}$, $C^* \approx 22$ mm$^{-3}$, $A^* \approx 34$ mm$^{-3}$. The NSFD scheme maintains positivity and accurately tracks the continuous solution.

### Example 3: effect of Beddington–DeAngelis parameters

**Fig. 4:**
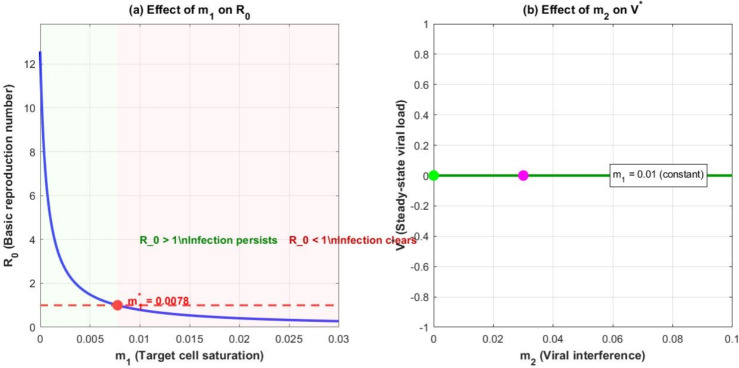
Effects of Beddington–DeAngelis parameters on infection dynamics. (a) Increasing $m_1$ reduces $\mathcal {R}_0$ and can lead to infection clearance when $\mathcal {R}_0 < 1$. When $m_1$ exceeds a critical value ($m_1 \approx 0.018$), $\mathcal {R}_0$ falls below 1. (b) Increasing $m_2$ reduces the endemic viral load but does not affect $\mathcal {R}_0$, consistent with Equation (5).

Figure 4 illustrates the effects of the B-D saturation parameters $m_1$ and $m_2$ on infection dynamics. Panel (a) shows that increasing $m_1$ (target cell saturation) reduces both $\mathcal {R}_0$ and the endemic viral load. When $m_1$ exceeds a critical value ($m_1 \approx 0.018$ in this example), $\mathcal {R}_0$ falls below 1 and the infection is cleared. Panel (b) shows that increasing $m_2$ (viral interference) reduces the endemic viral load but does not affect $\mathcal {R}_0$, consistent with Equation (5). This highlights the different roles of the two saturation parameters: $m_1$ affects whether infection can establish, while $m_2$ modulates the severity of established infection.

### Example 4: effect of immune response

**Fig. 5:**
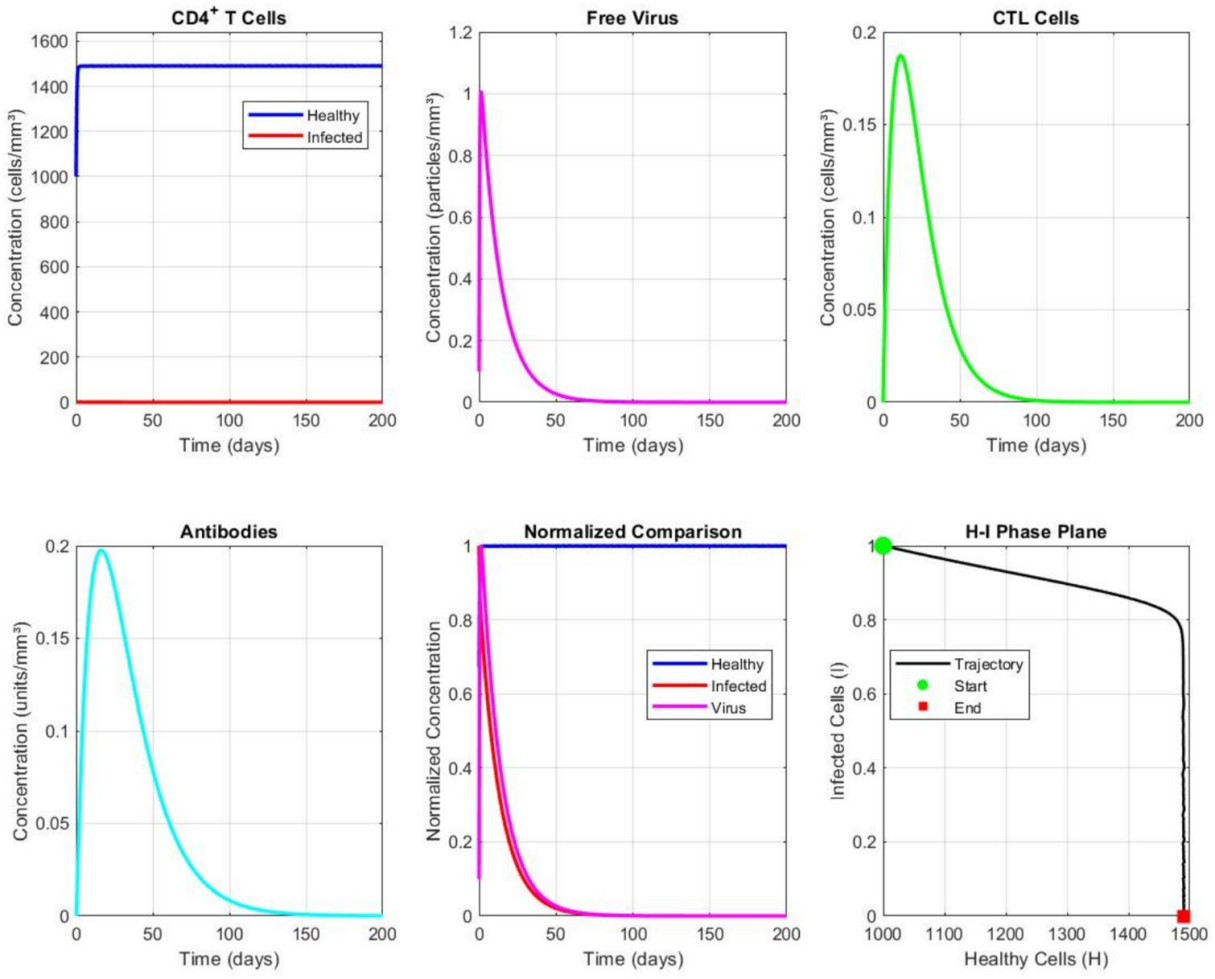
Effect of immune responses on HIV dynamics. (**a**) No immune response ($\lambda _C = \lambda _A = 0$): rapid disease progression with high viral loads. (**b**) Cellular immunity only ($\lambda _C = 0.05$, $\lambda _A = 0$): reduces viral load but does not prevent CD4$^+$ T-cell decline. (**c**) Humoral immunity only ($\lambda _C = 0$, $\lambda _A = 0.03$): partially controls viral load. (**d**) Both responses active: best control with higher CD4$^+$ T-cell counts and lower viral loads.

Figure 5 demonstrates the importance of immune responses in controlling HIV infection. Panel (a) shows the dynamics without immune responses ($\lambda _C = \lambda _A = 0$), resulting in high viral loads and rapid depletion of CD4$^+$ T-cells. Panel (b) shows the dynamics with only cellular immunity ($\lambda _C = 0.05$, $\lambda _A = 0$), which reduces viral load but does not prevent CD4$^+$ T-cell decline. Panel (c) shows the dynamics with only humoral immunity ($\lambda _C = 0$, $\lambda _A = 0.03$), which partially controls viral load. Panel (d) shows the dynamics with both immune responses active, resulting in the best control of infection with higher CD4$^+$ T-cell counts and lower viral loads.

### Example 5: bifurcation analysis

**Fig. 6:**
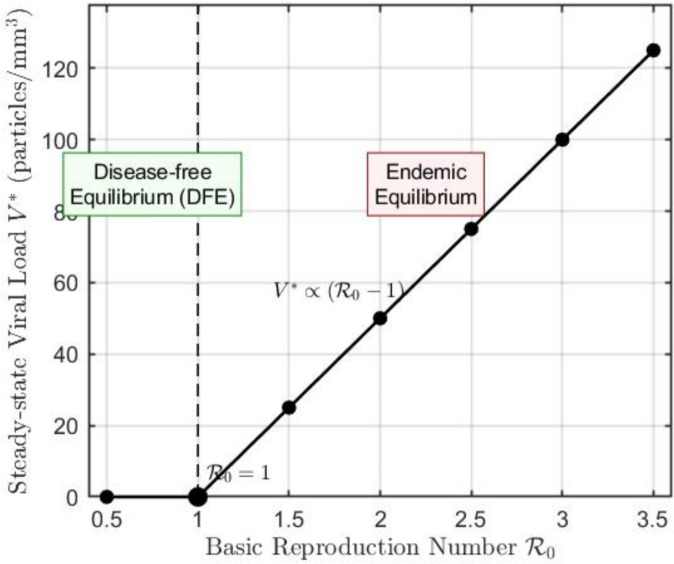
Bifurcation diagram showing the steady-state viral load $V^{*}$ as a function of the basic reproduction number $\mathcal {R}_0$. A transcritical bifurcation occurs at $\mathcal {R}_0 = 1$, where the disease-free equilibrium loses stability and the endemic equilibrium emerges. For $\mathcal {R}_0> 1$, $V^{*}$ increases with $(\mathcal {R}_0 - 1)$.

Figure 6 shows a bifurcation diagram with $\mathcal {R}_0$ as the bifurcation parameter (varied by changing $\beta$). As predicted by the theoretical analysis, a transcritical bifurcation occurs at $\mathcal {R}_0 = 1$: the disease-free equilibrium loses stability and the endemic equilibrium emerges. For $\mathcal {R}_0> 1$, the endemic equilibrium is stable, with the steady-state viral load increasing with $\mathcal {R}_0$. Across the biologically relevant parameter ranges explored in our simulations, we did not observe backward bifurcation. A rigorous analysis using center manifold theory would be required to definitively rule out backward bifurcation, which remains a direction for future investigation.

## Discussion and conclusion

This study has presented a comprehensive analysis of a within-host HIV dynamics model incorporating Beddington–DeAngelis incidence, cure rate, logistic growth, and immune responses. We now discuss the significance of our findings, compare them with existing literature, and suggest directions for future research.

### Summary of key findings

Our analysis yields several important theoretical and numerical results:

1. **Threshold Dynamics:** The basic reproduction number $\mathcal {R}_0$, given by Equation (5), serves as a sharp threshold determining infection outcome. If $\mathcal {R}_0 \le 1$, the disease-free equilibrium is globally asymptotically stable and infection clears. If $\mathcal {R}_0> 1$, a unique endemic equilibrium exists and is globally asymptotically stable, indicating persistent infection.
2. **Role of Saturation Parameters:** The Beddington–DeAngelis parameters $m_1$ and $m_2$ play distinct biological roles. The target cell saturation parameter $m_1$ reduces $\mathcal {R}_0$ and can potentially drive it below 1, leading to infection clearance. The viral interference parameter $m_2$ reduces the endemic viral load without affecting the threshold, modulating disease severity.
3. **Importance of Immune Responses:** Both cellular and humoral immune responses contribute to controlling HIV infection. Cellular immunity (CTLs) directly kills infected cells, while humoral immunity (antibodies) neutralizes free virus. Their combined action provides the most effective control, highlighting the importance of vaccines that elicit both types of responses.
4. **Dynamically Consistent Discretization:** The NSFD scheme preserves all essential qualitative features of the continuous model, including positivity, boundedness, and stability of equilibria. This provides a reliable numerical tool for exploring model behavior across different parameter regimes.

### Comparison with existing literature

Our model extends previous work in several important ways:

- Compared to models with bilinear incidence^3^, our model with B-D incidence predicts lower infection rates at high viral loads, which may better reflect biological reality where saturation effects occur.
- Compared to models with only cure rate^9^ or only immune response^12^, our unified framework allows investigation of how these mechanisms interact. We find that cure rate and immune responses can synergize to control infection more effectively than either alone.
- Compared to models without logistic growth^3^, our model with density-dependent proliferation provides a more realistic representation of CD4$^+$ T-cell homeostasis, which is important for long-term dynamics.
- The global stability results extend local stability analyses in previous studies^5,17^, providing stronger guarantees about long-term behavior.

### Biological implications and potential applications

Our findings have several implications for understanding and managing HIV infection:

1. **Therapeutic Strategies:** The model suggests that interventions can target different aspects of infection dynamics. Reducing $\beta$ (infection rate) or increasing $m_1$ (target cell saturation) lowers $\mathcal {R}_0$ and can lead to eradication. Increasing $\rho$ (cure rate), $m_2$ (viral interference), or immune parameters lowers the endemic viral load, corresponding to a “functional cure” with controlled viremia.
2. **Vaccine Design:** The importance of both cellular and humoral immunity suggests that effective HIV vaccines should aim to elicit both CTL and antibody responses. Our model provides a framework for quantifying the relative contributions of these responses.
3. **Personalized Medicine:** By fitting the model to patient data, one could estimate individual parameters (e.g., $\mathcal {R}_0$, immune responsiveness) and tailor treatment accordingly. Patients with high $\mathcal {R}_0$ or weak immune responses might require more aggressive therapy.
4. **Drug Development:** The model can be used to simulate the effects of new drugs targeting specific steps in the viral life cycle. For example, entry inhibitors effectively increase $m_1$, while neutralizing monoclonal antibodies increase $\kappa$.

### Limitations and future work

While our model advances understanding of HIV dynamics, it has limitations that suggest directions for future research:

1. **Simplified Immune Dynamics:** The model uses linear activation terms for immune responses. More realistic saturation functions (e.g., Michaelis-Menten) could be incorporated.
2. **Lack of Viral Diversity:** The model treats virus as homogeneous, while in reality HIV exhibits high genetic diversity and escape mutations. Incorporating viral evolution and immune escape would increase realism.
3. **Absence of Latent Reservoir:** The model does not include latently infected cells, which are crucial for viral persistence and rebound after treatment interruption.
4. **Constant Parameters:** Biological parameters may vary over time due to circadian rhythms, treatment adherence, or disease progression. Time-varying or stochastic parameters could be considered.
5. **Spatial Heterogeneity:** The model assumes well-mixed populations, neglecting spatial structure in lymphoid tissues. Partial differential equation or agent-based models could address this.
6. **Clinical Validation:** While parameters are based on literature estimates, fitting the model to longitudinal patient data would strengthen its clinical relevance.

Future work will address these limitations, with particular focus on incorporating latent reservoirs and viral evolution. We also plan to develop methods for parameter estimation from clinical data and explore optimal control strategies for treatment scheduling.

### Conclusion

In conclusion, we have developed and analyzed a comprehensive within-host HIV model that integrates multiple biological mechanisms: Beddington–DeAngelis incidence, cure rate, logistic growth, and immune responses. The model exhibits threshold dynamics governed by $\mathcal {R}_0$, with global stability of equilibria ensuring predictable long-term behavior. The NSFD discretization provides a reliable numerical tool that preserves the model’s qualitative features. Our findings offer insights into HIV pathogenesis and control, with potential applications in treatment optimization and vaccine design. As mathematical models become increasingly sophisticated and integrated with clinical data, they will play an essential role in the quest to end the HIV pandemic.

## Data Availability

The data generated during this study are available from the corresponding author upon reasonable request.

## A MATLAB code for numerical simulations

This appendix provides the complete MATLAB code used for all numerical simulations in this study. The code implements both the continuous model (using ode45) and the NSFD discretization.
